# Computational insights into charge transfer across functionalized semiconductor surfaces

**DOI:** 10.1080/14686996.2017.1370962

**Published:** 2017-09-26

**Authors:** Kara Kearney, Angus Rockett, Elif Ertekin

**Affiliations:** a Department of Materials Science and Engineering, University of Illinois, Urbana, Illinois, USA; b Department of Metallurgy and Materials Science, Colorado School of Mines, Golden, Colorado, USA; c Department of Mechanical Science and Engineering, University of Illinois, Urbana, Illinois, USA; d International Institute for Carbon Neutral Energy Research (WPI-I2CNER), Kyushu University, Fukuoka, Japan

**Keywords:** Density functional theory, device simulations, functionalized semiconductors, charge transfer, passivation layers, organic functionalization, photoelectrochemical water-splitting, multiscale modeling, photoelectrodes, 50 Energy Materials, 201 Electronics / Semiconductor / TCOs, 212 Surface and interfaces, 401 1st principle calculations, 402 Multi-scale modeling, 209 Solar cell / Photovoltaics, 206 Energy conversion / transport / storage / recovery

## Abstract

Photoelectrochemical water-splitting is a promising carbon-free fuel production method for producing H_2_ and O_2_ gas from liquid water. These cells are typically composed of at least one semiconductor photoelectrode which is prone to degradation and/or oxidation. Various surface modifications are known for stabilizing semiconductor photoelectrodes, yet stabilization techniques are often accompanied by a decrease in photoelectrode performance. However, the impact of surface modification on charge transport and its consequence on performance is still lacking, creating a roadblock for further improvements. In this review, we discuss how density functional theory and finite-element device simulations are reliable tools for providing insight into charge transport across modified photoelectrodes.

## Graphical abstract

**Figure:**
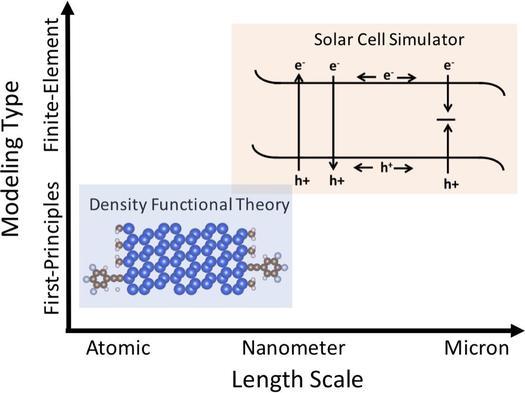

## 1. Introduction

Photovoltaic and photoelectrochemical (PEC) cells are two devices capable of harvesting the solar power incident on Earth by converting sunlight into electric power and/or chemical fuels [1]. High conversion efficiencies recently observed in PEC cells have motivated research to move from conventional photovoltaics towards a new generation of PEC cells [2,3]. These cells utilize a semiconductor photoelectrode to capture and convert incident light into free charge carriers that are exploited to provide the electrochemical difference required to generate chemical fuels. A promising carbon-free fuel-production method is PEC solar-driven water-splitting where hydrogen (H_2_) and oxygen (O_2_) gas are generated from liquid water [4–15].

**Figure 1.**
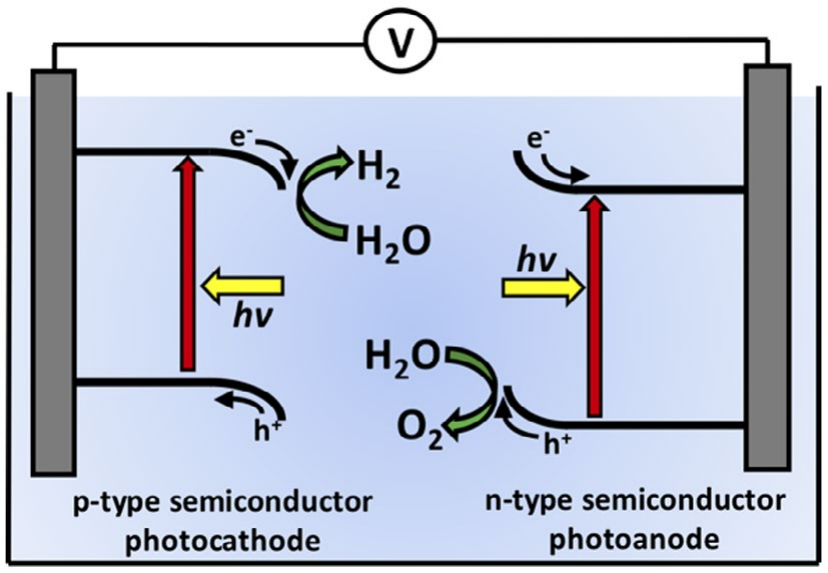
Schematic of a two-electrode photoelectrochemical water-splitting cell where a p-type semiconductor operates as the photocathode to evolve hydrogen while an n-type semiconductor operates as a photoanode to evolve oxygen.

A 1.23 V electrochemical difference is required as a minimum to drive PEC water-splitting, which can only be provided by a single semiconductor with an excessively large band-gap. An alternative strategy is to use two small band-gap semiconductors placed electrically in series. One semiconductor operates as the photocathode to drive H_2_-evolution while the other operates as the photoanode to drive O_2_-evolution, as shown in Figure 1. In this case, the electrochemical potential difference required to split water is partially provided by each semiconductor. However, small band-gap semiconductors typically dissolve or develop insulating oxide coatings during PEC operation. Current research is focused on developing various techniques for stabilizing small band-gap semiconductors for use in a technologically viable, efficient, and stable solar-driven water-splitting system.

**Figure 2.**
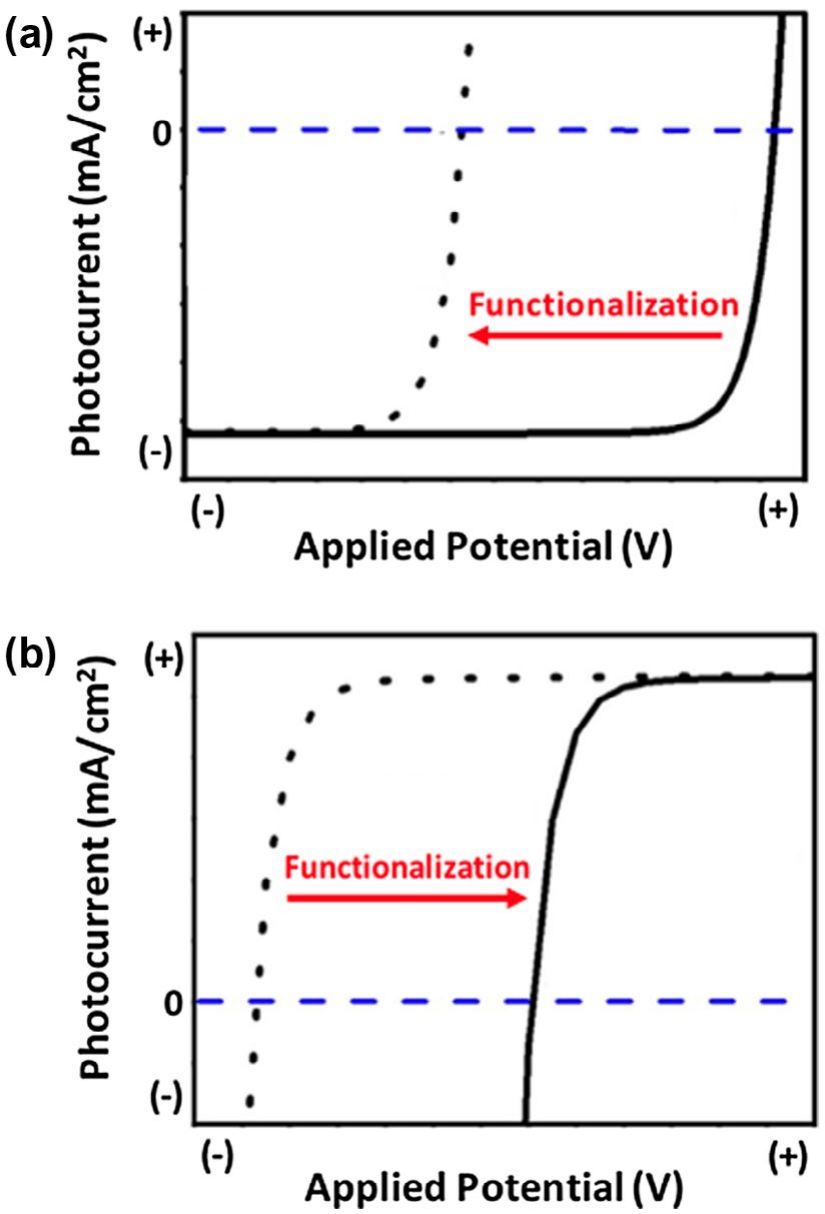
Commonly observed impact of surface functionalization on the photocurrent versus voltage response of a (a) p-type semiconductor photocathode and (b) n-type semiconductor photoanode.

Surface modification is commonly used in PEC cells [31,32] as well as a variety of additional applications such as electronic devices [16–21], data storage [22], chemical sensing [23–25], molecular nanopatterning [26], and bioengineering [27–30]. In PEC applications, chemical attachment of organic molecules is a versatile technique used to enhance the stability of low-gap semiconductors while controlling the physiochemical properties of the surface [33]. For example, Lewis et al. have shown that forming covalent Si-C bonds on the surface of Si(111) photoelectrodes prevents oxidation of the surface [34]. Alternatively, depositing a thin layer of metal oxide such as SiO_2_ [35], Al_2_O_3_ [36,37], TiO_2_ [38–42], or ZnO [43] has been shown to passivate a variety of semiconductor surfaces. Although functionalizing a semiconductor with organic molecules or passivation layers stabilizes the surface, the modification often results in a less preferable onset potential of the semiconductor photoelectrode as shown for a p-type photocathode (more negative) and n-type photoanode (more positive) in Figure 2(a) and Figure 2(b), respectively.

An understanding of how surface modification influences charge transport and reduces the onset potential of a functionalized semiconductor photoelectrode is still lacking. This creates a roadblock for further improvements in device performance and a lack of design rules for identifying candidate materials for functionalization. In this review, we discuss how computational modeling can be used to provide insight into charge transfer across heterojunction structures. Specifically, we show examples of applying first-principles density functional theory and finite-element device simulations to investigate the effects of organic functionalization and passivating layers on charge transport across an underlying semiconductor surface.

## 2. Experimental methods

### 2.1. Photocurrent versus voltage response

Photoelectrode performance can be quantified experimentally by measuring the photocurrent density versus voltage (J-V) response for a specified potential range under solar illumination in contact with a redox couple. Two benchmarks directly related to the performance are obtained from the J-V curve: (1) short-circuit current density (J_sc_), which is the maximum observed current at 0 V applied potential and (2) onset potential for the redox reaction (V_on,A_), which is the voltage at which the net current across the device is zero.

The onset potential is a key metric of performance for photoelectrochemical devices because it is related to the open-circuit photovoltage, V_oc_, which is the maximum energy that can be obtained from the collection of photons to drive electrical current and/or a chemical reaction. The V_oc_ can be calculated from V_on,A_ using [44]

(1) $${\text{V}}_{\text{oc}}=\text{E}\left(\text{A}/{\text{A}}^{-}\right)-{\text{V}}_{\text{on,A}}$$

where E(A/A^-^) is the formal redox potential of the redox couple in contact with the photoelectrode. The maximum V_oc_ that can be achieved for a device is limited by recombination processes in the bulk of the material and can calculated for a p-type semiconductor as [45]

(2) $${\text{V}}_{\mathrm{oc}}\;=\;\frac{\mathrm{kT}}{q}ln\left(\frac{{\text{J}}_{\text{ph}}{\text{L}}_{\text{n}}}{{\text{qN}}_{\text{c}}{\text{D}}_{\text{n}}}\right)+\frac{E_{g}}{q}-V_{p}$$

where J_ph_ is the photocurrent density, L_n_ is the minority carrier diffusion length, D_n_ the minority carrier diffusion coefficient, N_c_ the effective density of states in the conduction band, and V_p_ the difference in energy between the Fermi and valence band energies in the bulk. A similar equation can be written for an n-type semiconductor. A V_oc_ less than the value calculated by Equation (2) is a consequence of deleterious surface or bulk effects in the semiconductor such as poor diffusion lengths, thermionic emission or tunneling across heterojunctions or surface barriers, transport through chemical imperfections, surface/bulk state recombination, or Fermi level pinning [45, 46].

### 2.2. Dipole measurements

**Figure 3.**
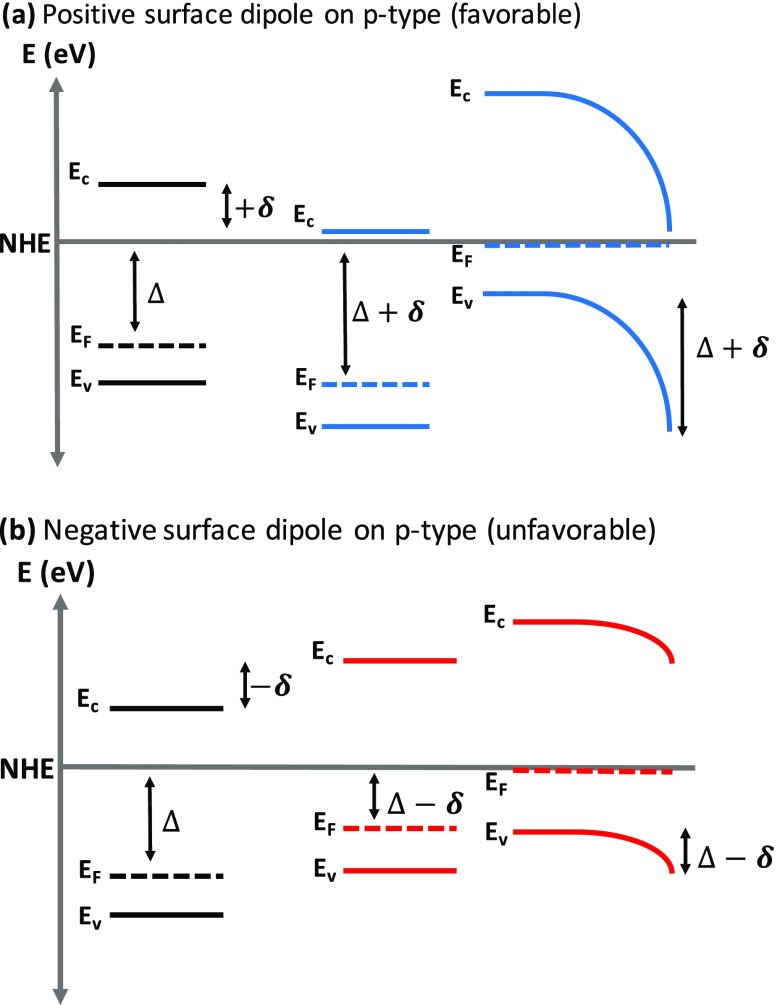
The effect of a (a) positive (favorable) and (b) negative (unfavorable) surface dipole on the band bending and depletion width of a p-type semiconductor.

Functionalization of a photoelectrode with organic molecules has been observed to dramatically affect the V_oc_ due to a change in the magnitude and direction of the surface dipole induced by the organic molecule. For a p-type semiconductor, a molecule that induces a large positive dipole (negative charge on the molecule, positive charge on the substrate) increases the V_oc_ by increasing the barrier height and depletion width at the surface as shown in Figure 3(a), while a negative dipole (positive charge on the molecule, negative charge on the substrate) decreases the barrier height and depletion width as shown in Figure 3(b). For an n-type semiconductor the behavior is reversed and a molecule with a negative dipole increases the V_oc_ by increasing the barrier height and depletion width at the surface.

The surface dipole (δ) is defined as the difference between the electron affinity at the surface (χ_s_) and the electron affinity in the bulk (χ_B_) of the semiconductor

(3) $$\delta =\chi_{s}-\chi_{B}.$$

*χ*_*s*_ can be expressed in terms of the work function at the surface (Φ_s_), band gap of the semiconductor (E_g_), and the absolute energy difference between the valence band edge and the Fermi level (|E_v_−E_F_|_s_) at the surface resulting in an expression for the surface dipole as

(4) $$\delta =\Phi_{S}-E_{g}+{\left|{\text{E}}_{\text{V}}-{\text{E}}_{\text{F}}\right|}_{S}-\chi_{B}.$$

Equation (4) allows for a direct determination of the surface dipole because the values of E_g_ and *χ*_*B*_ can be found in literature while the magnitude of $\Phi_{S}$ and |E_v_−E_F_|_s_ can be measured from ultraviolet photoelectron spectroscopy (UPS) and X-ray photoelectron spectroscopy data (XPS), respectively [47–49].

**Figure 4.**
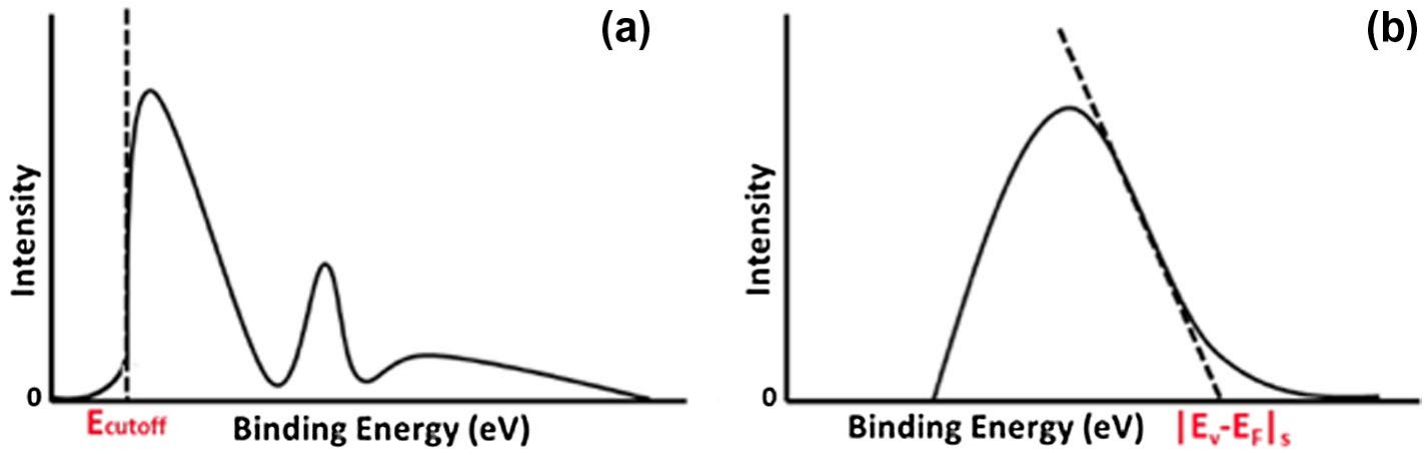
(a) Schematic of ultraviolet photoelectron emission data with the value of the cutoff energy indicated on the plot. (b) Schematic of X-ray photoelectron spectroscopy data with the value of the absolute energy difference between the valence band-edge and the Fermi level (|E_V_- E_F_|_s_) at the surface indicated on the plot.

The work function of a semiconductor can be determined using UPS via the following expression

(5) $$\Phi s=\;h\upsilon -{\text{E}}_{\text{cutoff}}$$

where *hv* is the excitation energy (typically He I emission at 21.2 eV) and E_cutoff_ is the secondary electron cutoff energy in the photoelectron spectra [50]. The secondary emission is due to secondary electrons, which are defined as those that have lost energy due to inelastic collisions when traveling to the sample surface. The secondary emission is characterized by a broad, continuous peak. E_cutoff_ is determined graphically by extrapolating the secondary electron cutoff energy to the background intensity as shown in Figure 4(a). |E_V_−E_F_|_s_ can be determined using XPS with He II emission at 40.82 eV. The value of |E_V_−E_F_|_s_ is determined graphically by extrapolating the steep emission onset in the spectra to zero signal as shown in Figure 4(b). Once $\Phi_{s}$ and |E_V_−E_F_|_s_ are determined the sign and magnitude of the surface dipole can be calculated using Equation (4).

## 3. Theoretical methods

**Figure 5.**
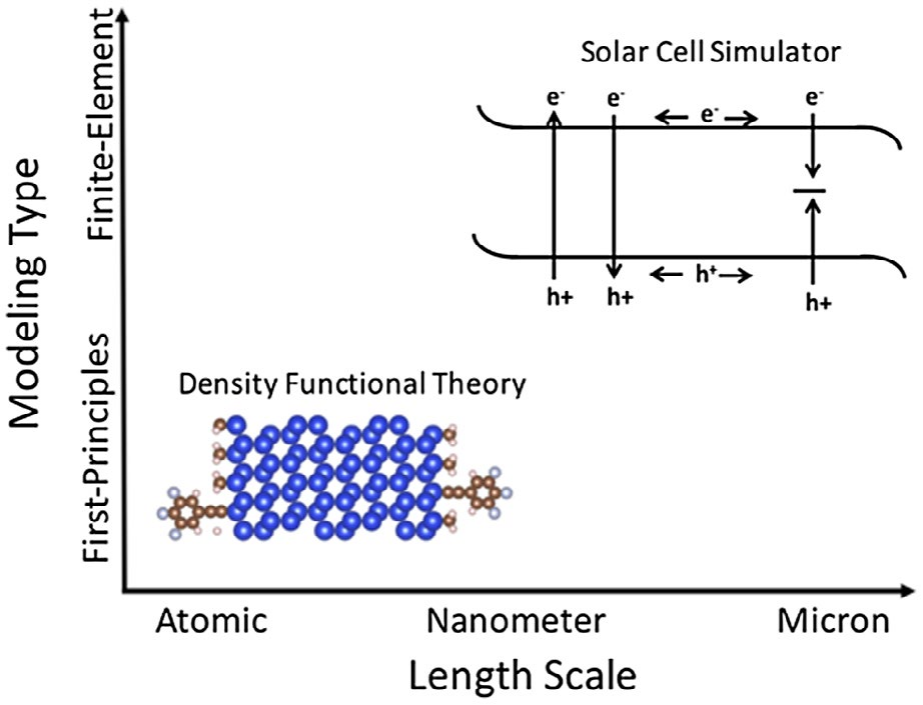
Schematic of the two computational methods described in this review. First-principles modeling is on the scale of electrons/atoms while device simulations are on the micron/device scale.

We demonstrate simulation-based improvement of charge transport across modified semiconductor surfaces by utilizing two independent computational methods: first-principles density functional theory [51,52] and a finite element-based device simulator known as wxAMPS [53]. Density functional theory is utilized to investigate the electronic properties of the semiconductor surface at the atomic level while wxAMPS provides insight into the performance of the semiconductor photoelectrode at the device level. Utilizing both computational methods provides a theoretical tool for analyzing the system from the atomic to the device level as shown in Figure 5.

### 3.1. Density functional theory

Density functional theory (DFT) is a quantum mechanical modeling method applicable to atomic scale systems and widely employed in the field of computational chemistry and physics. DFT is used to investigate the electronic structure of many-body systems by solving the Schrödinger equation to yield the total energy (E) of a system

(6) $$H\psi \left(\vec{r_{i}},\vec{R_{j}}\right)=E\psi \left(\vec{r_{i}},\vec{R_{j}}\right)$$

where ***H*** is the Hamiltonian operator and *ψ* is the wavefunction depending on the spatial coordinates of electrons, $\vec{r_{i}}$, and nuclei, $\vec{R_{j}}$, in the system.

Equation (6) represents a many-body system with N electrons and 3 N total spatial coordinates, which is computationally intractable. Therefore, an approximation known as Kohn-Sham DFT is often used in which the system is approximated as a collection of non-interacting electrons moving in an effective potential, V_eff_. Equation (6) then becomes a one-electron Schrödinger equation for a 3-coordinate system described by Equation (7)

(7) $$H\left(V_{\mathrm{eff}}\left(\vec{r}\right)\right)\psi_{i}\left(\vec{r}\right)=\varepsilon_{i}\psi_{i}\left(\vec{r}\right)$$

where *ε*_*i*_ is the energy eigenvalue corresponding to the Kohn-Sham one-electron wavefunction, $\psi_{i}\left(\vec{r}\right)$. The charge density, $\rho (\vec{r})$, for the N-electron system is obtained as a sum over occupied states by Equation (8)

(8) $$\rho \left(\vec{r}\right)=\;\sum_{i}^{N}{\left|\psi_{i}\left(\vec{r}\right)\right|}^{2}\;.$$

The use of Kohn-Sham DFT requires an approximate exchange-correlation functional chosen to represent the spatial dependence of the effective potential. The accuracy of DFT in modeling various systems is limited by the chosen functional for the system. However, because DFT is often used to calculate the energy difference between various systems, errors due to the exchange-correlation functional often cancel leading to reliable results.

The most commonly chosen functionals are LDA [54] and PBE [55] due to their computational reliability and low computational cost. Nowadays, more sophisticated options are available such as hybrid functionals, however, these are often still computationally limiting. Many codes are available to compute DFT calculations including VASP [56–60], Quantum Espresso [61], and SIESTA [62].

#### 3.1.1. Modeling surfaces

**Figure 6.**
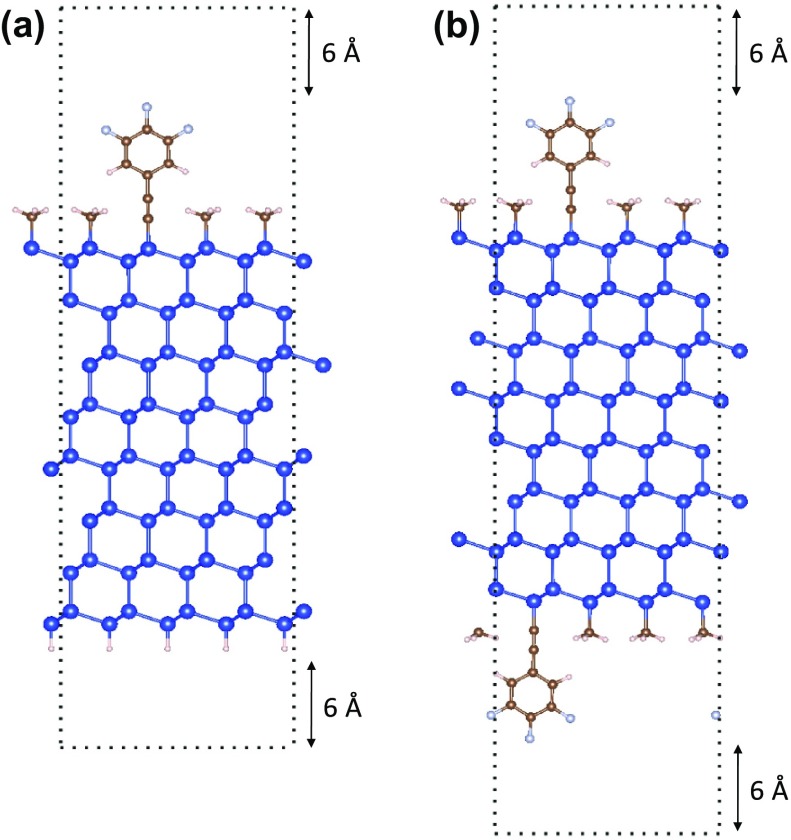
(a) Asymmetric slab configuration where the top side of the slab is terminated with a mixed monolayer of trifluorophenylacetylenyl and methyl groups while the bottom side is passivated with hydrogen. (b) Symmetric slab configuration where the top and bottom sides of the slab are terminated with a mixed monolayer of trifluorophenylacetylenyl and methyl groups. In both configurations 12 Å of vacuum separates the surfaces of the slab. The dark blue, brown, light pink, and light blue atoms represent silicon, carbon, hydrogen, and fluorine, respectively.

First-principles calculations based on DFT have become a powerful tool for calculating surface properties of semiconductors [63–72]. Surface calculations using DFT are often computed using a planewave basis [73] and a slab configuration (see Figure 6). The slab is placed within a 3D periodic super-cell and a vacuum layer is added on either side of the slab to mimic the 2D periodicity of an actual surface. The number of slab layers should be thick enough to minimize surface-surface interactions and recover a bulk-like interior. Similarly, the vacuum layer is thick enough such that interactions between the two sides of the slab or the formation of a net dipole are avoided.

A slab may have an asymmetric (Figure 6(a)) or symmetric (Figure 6(b)) configuration which are defined as having different or identical terminations on the two sides of the slab, respectively. When using an asymmetric slab, an artificial electric field is automatically added to the cell to eliminate the potential step resulting from a difference in the two sides of the slab. This field may artificially change the electronic structure of the absorbed monolayer resulting in qualitative as well as quantitative errors.

An established method to correct for the artificial electric field is to place a dipolar sheet inside the vacuum region such that the total dipole across the super cell is zero [74]. However, we and others have found that this correction scheme often results in convergence problems [70]. To avoid the convergence issues associated with calculating the total dipole for an asymmetric slab, we recommend using a symmetric slab when possible because the total dipole for a symmetric slab is zero, which eliminates the artificial electric field. In some situations, it is necessary to use an asymmetric slab, such as for polar/non-centrosymmetric crystals with opposite terminations. In this case, a very large vacuum region may be used (30+ Å) to mitigate the magnitude of the electric field interacting with the surface but convergence of properties with respect to vacuum length is slow.

#### 3.1.2. Quantifying a surface dipole

**Figure 7.**
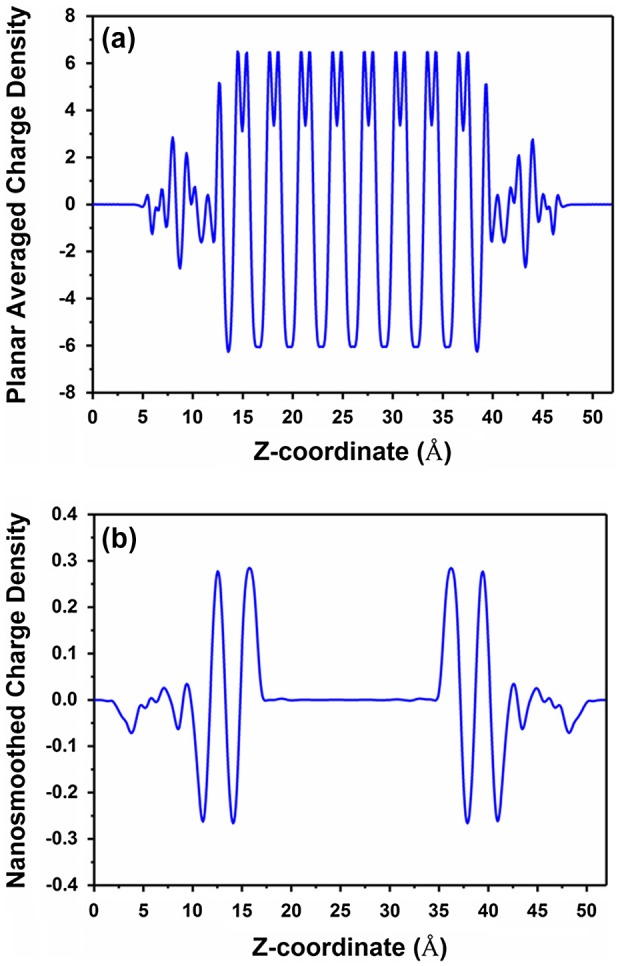
Planar average charge density and nanosmoothed average charge density for the symmetric terminated Si(111) slab configuration shown in Figure 6(b).

The interfacial dipole of a semiconductor functionalized with an arbitrary functional group can be calculated using DFT by a procedure known as nanosmoothing developed by Rabe et al. [75]. Nanosmoothing is used to eliminate bulk effects and extract well-defined, unique values of the interfacial charge and dipole densities for a system. The procedure involves calculating a smoothed charge density, $\overset{¯}{\rho }(z)$, in which periodic bulk oscillations in the microscopic charge density are removed by convoluting the planar-averaged charge density calculated in Equation (9) with a smoothing function f(z) as shown in Equation (10) [74, 76]

(9) $$\rho \left(z\right)=\frac{1}{A}\overset{A}{\underset{0}{\;\int \int \;}}\rho \left(x,y,z\right)\text{d}x\text{d}y$$

(10) $$\overset{¯}{\rho }\left(z\right)=\int^{}\rho \left(z^{{}^{\prime }}\right)f\left(z-z^{{}^{\prime }}\right)\text{d}z^{\prime }$$

where A is the surface area of the slab in the z-direction. The planar-average charge density and the nanosmoothed charge density are shown in Figure 7(a) and 7(b), respectively, for the symmetric terminated Si(111) slab configuration shown in Figure 6(b).

The smoothed charge density is a continuous function that approaches zero at regions far from the interface. The interface is defined as the region where the smoothed charge density deviates away from zero. The nanosmoothed dipole density, $\overset{¯}{\text{p}}$, is unique and well defined if z_1_ and z_2_ are in regions where the smoothed charge density is zero and is calculated using

(11) $$\overset{¯}{\text{p}}=\int_{z_{1}}^{z_{2}}\overset{¯}{\rho }\left(z\right)z\text{d}z$$

### 3.2. Finite-element device simulations

wxAMPS is a drift-diffusion solid-state device simulation application based on AMPS, a solar cell simulation program developed by Fonash et al. at Pennsylvania State University [77]. wxAMPS has the same user inputs and physical principles as AMPS but incorporates a more advanced algorithm combining the Newton and Gummel methods [78] to facilitate convergence and improved models of recombination and tunneling [79,80]. wxAMPs is available free of charge on the web and has an easy-to-use graphical user interface [81]. wxAMPS has been demonstrated to model a variety of complex devices including dye-sensitized solar cells [82] and functionalized photoelectrodes [83,84].

wxAMPS simulates charge generation, recombination, and transport across single or multijunction devices by solving Poisson’s equation, the continuity equation for free holes, and the continuity equation for free electrons simultaneously. wxAMPS uses the one-dimensional form of Poisson’s equation to relate the charge carrier concentrations throughout the device to the electrostatic potential, V, referenced to the position of the local vacuum level:

(12) $$\frac{d}{\mathrm{dx}}\left(\;\varepsilon \left(x\right)\frac{\mathrm{dV}}{\mathrm{dx}}\right)=q*\left[p\left(x\right)-n\left(x\right)+N_{D}^{+}\left(x\right)-N_{A}^{-}\left(x\right)+p_{t}\left(x\right)-n_{t}\left(x\right)\right]$$

where n, p, n_t_, p_t_, N_D_^+^, N_A_^-^ are the free electron, free hole, trapped electron, trapped hole, ionized donor, and ionized acceptor state concentrations as a function of position x in the device. *ɛ* is the permittivity of the material and q is the magnitude of the charge of an electron. The continuity equations for free electrons and holes in the delocalized states of the conduction band and valence band are given by Equations (13) and (14)

(13) $$\frac{1}{q}\left(\frac{\text{d}J_{\text{n}}}{\text{d}x}\right)=\;-G_{\mathrm{op}}\left(x\right)+R\left(x\right)$$

(14) $$\frac{1}{q}\left(\frac{\text{d}J_{\text{p}}}{\text{d}x}\right)=G_{\mathrm{op}}\left(x\right)-R\left(x\right)$$

where J_n_ and J_p_ are the electron and hole current densities, respectively. R(x) is the net recombination rate from band-to-band and/or via Shockley-Read-Hall recombination, while G_op_(x) is the optical generation rate from illumination. A more detailed description of the model and the implementation of boundary conditions is described in previous work [83].

## 4. Application of computational methods

### 4.1. Organic functionalization

Chemical attachment of organic molecules to a semiconductor surface may alter the direction and magnitude of the dipole at the surface [32]. We describe here how density functional theory and wxAMPS can be combined to quantify the dipole of an arbitrary functionalization and the methods are then compared with experimental measurements.

#### 4.1.1. Band-edge control of silicon

Galli et al. conducted a combined theoretical and experimental study on the band-edge control of functionalized silicon [71]. Theoretical methods included electronic structure calculations using DFT and many-body perturbation theory (MBPT), while experimental methods included photoelectron spectroscopy and electrical device measurements. In their work, the ionization potential (IP), defined as the energy of the maximum valence band-edge relative to vacuum, of -H, -CH_3_, -C_2_H_5_, -Cl, and -Br terminated Si(111) surfaces were compared.

The IPs were determined experimentally by measuring the work function using ultraviolet photoelectron spectroscopy and calculating the IPs from

(15) $$\text{IP}=WF+\left(E_{F}-E_{\mathrm{vb}}\right)-\left(E_{\mathrm{vs}}-E_{\mathrm{vb}}\right)$$

where E_vb_ and E_vs_ are the bulk and surface valence-band levels, respectively. E_cb_ – E_F_ is calculated from the dopant density and E_bb_ is derived via a methodology described in Ref [48]. The IPs were calculated using DFT with local density approximation (LDA) exchange potentials and MBPT with the perturbative G_0_W_0_ approach.

At the LDA level, the calculated IPs were underestimated by 0.5–0.7 eV compared to the experimental results, while the values improved significantly using G_0_W_0_. However, the relative shifts in the IP between a given functionalization and Si(111)-H, ΔIP_*R*:*H*_, were the same for both LDA and GW and also in satisfactory agreement with experiments. Therefore, they suggested that LDA is adequate for predicting the absolute value for IP, IP^pred^, without resorting to expensive GW calculations. This is done by calculating the shift between a given functionalization and a well-defined reference state, $\Delta {\text{IP}}_{R:ref}^{\mathrm{DFT}}$, and using

(16) $${\text{IP}}^{\mathrm{pred}}\approx \;\Delta {\text{IP}}_{R:ref}^{\mathrm{DFT}}+{\text{IP}}^{\exp}\left(\mathrm{ref}\right)$$

where ${\text{IP}}^{\exp}\left(\mathrm{ref}\right)$ is the experimental known value of the IP for the reference state used to calculate $\Delta {\text{IP}}_{R:ref}^{\mathrm{DFT}}$.

Shifts in the IP are a consequence of a shift in the surface potential, Δ*V*_*surf*_, which is related to the formation of a surface dipole normal to the surface plane of the unit cell, $\mu_{surf,z}$, by

(17) $$\Delta V_{\mathrm{surf}}=\frac{-4\pi \mu_{surf,z}}{A}$$

*μ*_*surf*,*z*_ can be quantified as the sum of the dipole moment formed in the adsorbate radical, $\mu_{R,z},$ and the induced dipole moment due to charge transfer between the substrate and the absorbed molecule, $\mu_{ind,z}$. To gain an understanding of how the magnitude of $\mu_{surf,z}$ depends on the composition and coverage of the adsorbate, Galli et al. computed $\mu_{surf,z}$ and $\mu_{R,z}$ for a variety of terminal groups at both one-quarter and full coverage [71]. *μ*_*ind*,*z*_ was calculated using $\mu_{ind,z}$ = $\mu_{surf,z}-$$\mu_{R,z}$. They found that for non-polar terminal groups, $\mu_{R,z}=0$, trends in electronegativity dictated the charge exchange at the adsorbate/substrate interface and hence could be used to predict the magnitude of $\mu_{surf,z}$. They found that for polar terminal groups the magnitude of $\mu_{surf,z}$ depends on the orientation and surface coverage of the adsorbate, strength of intermolecular screening, and molecular polarizability.

**Figure 8.**
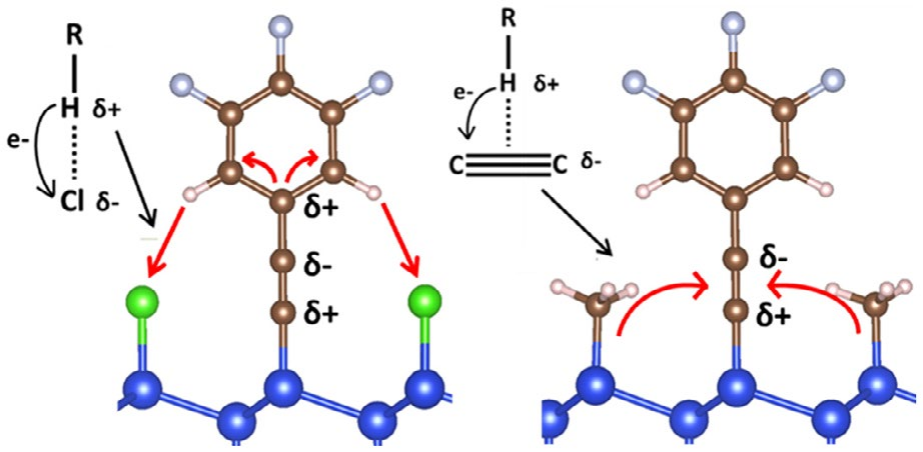
Lateral interactions are observed between neighboring moieties on mixed monolayers of Si(111) functionalization with trifluorophenylacetylene (TFPA) and -CH_3_/Cl. Reprinted with permission from *J. Phys. Chem. C*, *121*, 11312–11318. Copyright 2017 American Chemical Society [85].

In another study, the composition and coverage of mixed monolayers on Si(111) was found to have a significant effect on the interfacial dipole. The dipole as a function of coverage of 3,4,5-trifluorophenylacetylenyl (TFPA) moieties on Si(111) for both mixed methyl/TFPA and mixed chlorine/TFPA terminated surfaces was calculated using the nanosmoothing method discussed in section 3.1.2 [85]. The method was validated and the calculated trends were consistent with previously published experimental data. Significant interactions were found between neighboring species on the functionalized surface that significantly altered the magnitude of the interfacial dipole as shown in Figure 8. These results imply that the chemical species passivating non-bonded sites (i.e. -H, -Cl, -CH_3_, etc.) must match the experimental surface when predicting surface dipoles using DFT. These findings also suggest that the effective band-edge energies of a surface can be engineered to have a specific value by tuning the interfacial dipole with the chemical make-up of a mixed monolayer.

wxAMPS can also be used to predict the magnitude of an interfacial dipole on a functionalized semiconductor by fitting experimental data [83]. For example, Lewis et al. previously measured the V_oc_ for n and p-type Si(111)-H and Si(111)-CH_3_ photoelectrodes in contact with a series of redox couples in CH_3_CN-1.0 M LiClO_4_ [33]. Two different behaviors were observed for Si(111)-H and Si(111)-CH_3_. The magnitude of the surface dipole was predicted using wxAMPS by fitting the experimental trends. Because an interfacial dipole modifies the effective electron affinity of the surface of the semiconductor, the magnitude of the electron affinity was adjusted until the calculated trend in V_oc_ versus redox formal potential matched the experimental data. The results predicted a dipole shift of −0.35 eV between Si(111)-H and Si(111)-CH_3_ [83].

The dipole shift between Si(111)-H and Si(111)-CH_3_ was calculated in Galli’s work with DFT and many-body perturbation theory (MBPT) and found to be −0.8 eV in contact with vacuum [71]. The dipole shift determined using impedance spectroscopy in contact with octamethylferrocene-CH_3_CN-1.0 M LiClO4 is measured as −0.4 V and −0.25 V for n and p-Si(111), respectively [33]. The dipole shift determined using photoelectron spectroscopy in contact with vacuum is measured as −0.49 eV [48,49], [86,87]. These results are summarized in Table 1 below.

**Table 1.** Comparison of experimental and theoretical reports of the dipole shift between Si(111)-CH_3_ and Si(111)-H.

Experimental	Theoretical		
Impedance [33]	Photoelectron Spectroscopy [48,49], [86,87]	wxAMPS [83]	DFT & MBPT [71]
−0.40 V (n-Si)	−0.49 eV	−0.35 eV	−0.80 eV
−0.25 V (p-Si)	(p/n-Si)	(p/n-Si)	(p/n-Si)

As can be seen in Table 1 the dipole shift determined from wxAMPS is in better agreement with the experimental values than DFT and MBPT. In experiment, partial screening of a surface dipole occurs in the presence of an electrolyte. This effect is captured by wxAMPS because the value is determined by fitting experimental data directly. The resulting value includes both the raw dipole and the screening effects to yield the true final dipole of the complete system. The presence of the electrolyte is ignored when predicting the dipole using DFT and MBPT since the surface is simulated in contact with vacuum. Therefore, the difference between the values of the dipole determined by wxAMPS and DFT/MBPT provides insight into the degree of electrolyte screening occurring in a system (−0.45 eV in the example above). We note that the screening effect reduces the dipole by a factor of two or more. However, we have found that DFT is reliable for computing relative values of interfacial dipole.

#### 4.1.2. Organic-metal interfaces

Pennino et al. conducted a combined theoretical and experimental study on the work-function changes of metals functionalized by organic adsorbates [88]. Comparison between experiment and theory provided a clear picture of the role of molecular dipole moment, charge transfer, and Pauli repulsion on the work-function changes induced by functionalization. Theoretical methods included electronic structure calculations using DFT while experimental methods included photoelectron spectroscopy. The work-function changes of dimethyldisulfide, (CH_3_S)_2_ (DMDS), and methylthiolate, CH_3_S (MT) terminated Au(111) were compared. The work function changes were determined experimentally using UPS and theoretically by calculating the difference between the electrostatic potential in vacuum, V_vacuum_, and the Fermi level of the Au(111), E_Fermi_:

(18) $$\Phi \;=\;{\text{V}}_{\text{vacuum}}\;-\;{\text{E}}_{\text{Fermi}}$$

The experimentally observed work-function changes for DMDS and MT terminated Au(111) were found to be −1.2 and −1.5 to −1.7 eV, respectively, relative to the clean Au(111) surface, which is in good agreement with the calculated values of −1.2 and −1.6 eV, respectively.

**Figure 9.**
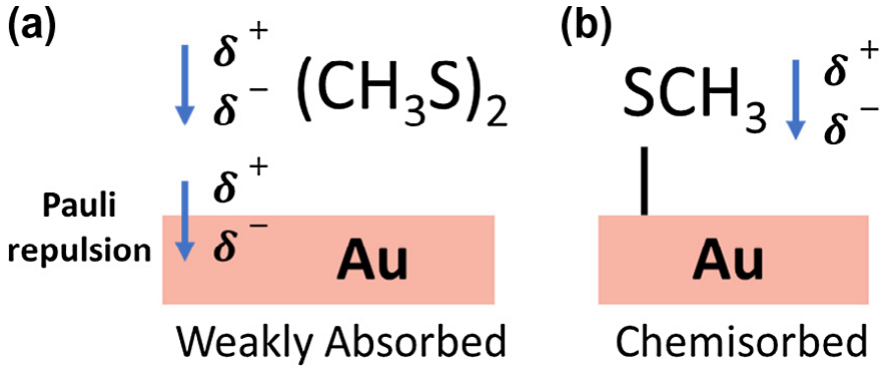
Comparison of (a) weakly absorbed DMDS on Au in which the main contributions to the work-function shift are the dipole intrinsic to the molecule and charge displacement via Pauli repulsion and (b) MT on Au in which the only contribution to the work-function shift is the dipole intrinsic to the molecule.

To determine the main mechanism for the observed work-function shift the electrostatic potential for the total system, V_total_, was compared with the sum of the electrostatic potential of the two subsystems, V_subsystems_, which consisted of the Au surface slab and molecular layer. For MT terminated Au(111), V_total_ − V_subsystems_ remains constant across the interface implying no charge transfer and that the dominant mechanism responsible for the work-function shift is the molecular dipole moment intrinsic to MT. This was confirmed by the good agreement between the calculated molecular dipole of the isolated MT layer and the experimental observation. For DMDS-terminated Au(111), V_total_ − V_subsystems_ decreases across the interface implying a transfer of electrons from the adsorbate layer to the Au substrate. Because the electron affinity between DMDS is the same as Au, the charge displacement must arise from compression of the Au surface charge density into the Au bulk due to Pauli repulsion with electrons in the DMDS layer. This comparison is illustrated in Figure 9.

### 4.2. Passivation layers

Coating a photoelectrode with a thin film metal oxide passivation layer is commonly employed to protect semiconductor surfaces against oxidation. However, metal oxide passivation layers may increase or decrease the onset potential of the electrode depending on the deposition conditions of the metal oxide and thus how charge is transferred across the heterostructure [42]. Javey et al. have observed that the photovoltage of *p*-InP photoelectrodes, which were passivated with TiO_2_ grown by atomic layer deposition (ALD), strongly depends on the type of titanium precursor used to grow the film. They found that in one case the TiO_2_ was ‘leaky’ and allowed significant hole conduction across the film resulting in a lower photovoltage. In an alternative case, the TiO_2_ was ‘non-leaky’ and hole conduction was blocked, resulting in a higher photovoltage [42].

We describe here how the link between the charge transport behavior and the magnitude and direction of the induced shift in open-circuit potential can be understood by using wxAMPS to model the generation, recombination, and charge transport across semiconductor/metal-oxide heterostructures.

#### 4.2.1. Leaky amorphous titanium dioxide

In semiconductor/metal-oxide heterostructures, a large valence band offset is usually observed between the wide-gap metal oxide and a relatively low-gap underlying semiconductor, which results in a barrier to hole transport across the photoelectrode. However, recent studies of amorphous TiO_2_ (‘*a*-TiO_2_’) grown by ALD using a nitrogen-based precursor resulted in a new ‘leaky dielectric’ passivation layer in which sufficient hole transport is observed through films hundreds of nanometers thick [89, 90]. The hole charge transport mechanism responsible for this leakage current is poorly understood experimentally due to the lack of methods available for probing such transport.

**Figure 10.**
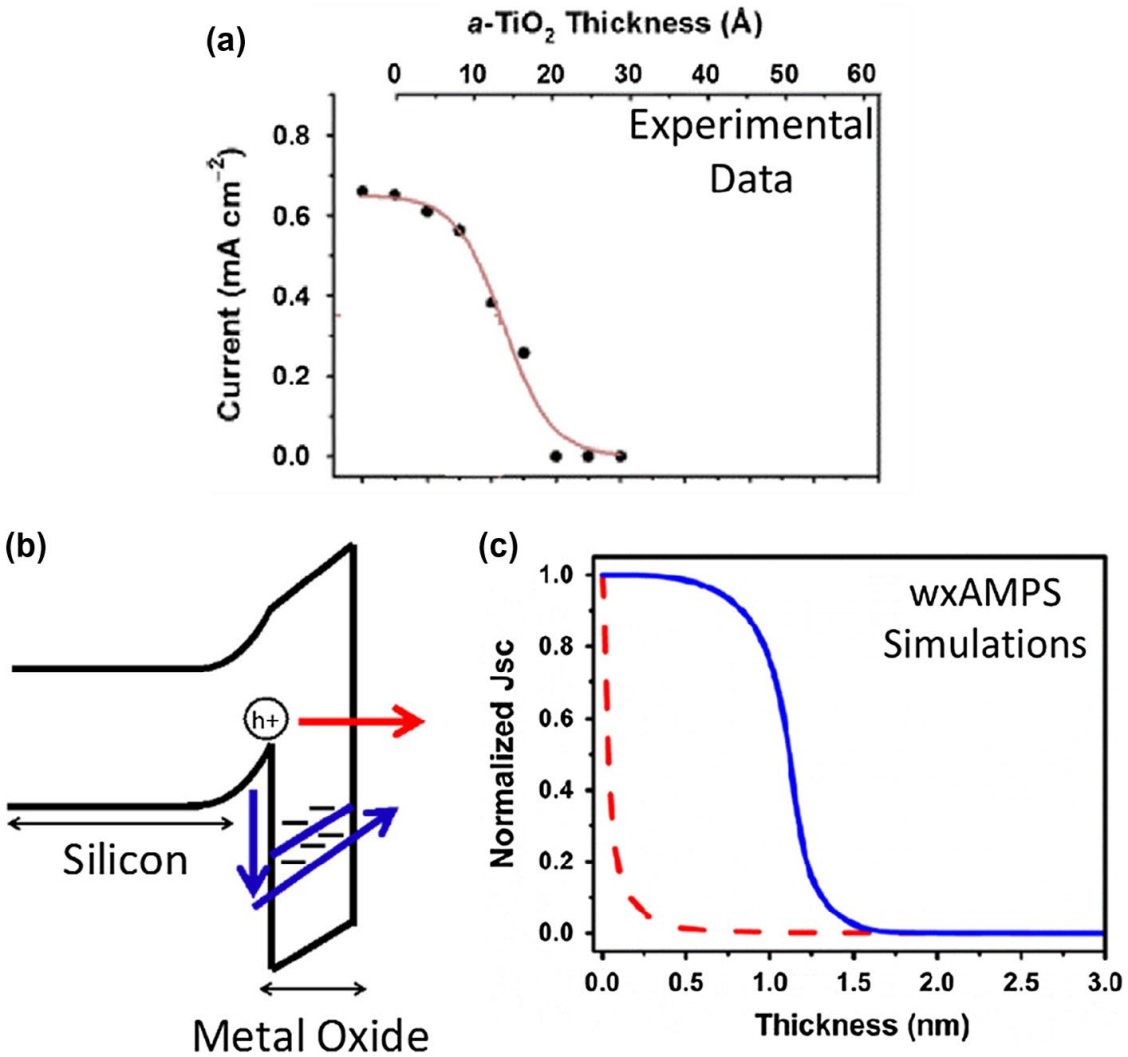
(a) Current versus TiO_2_ thickness behavior observed experimentally, (b) the two transport mechanisms simulated in wxAMPS: red arrow (tunneling), blue arrow (defect mediated transport), and (c) simulated current versus thickness behavior for the tunneling (red dashed) and defect-mediated transport (blue solid). Reprinted with permission from *J. Phys. Chem. C.*, *120* (45), 25697–25708, 2016. Copyright 2016 American Chemical Society [39].

wxAMPS has been used to investigate the ‘leaky’ dielectric transport mechanism. Rose et al. measured the anodic photocurrent of *n*-Si(111)-CH_3_|*a*-TiO_2_ electrodes as a function of *a*-TiO_2_ thickness [39]. They found that a maximum hole current was observed for sufficiently thin *a*-TiO_2_, decreasing beyond a critical thickness and resulting in negligible current as shown in Figure 10(a).

Two different hole conduction mechanism have been suggested for this observed behavior: tunneling and defect mediated transport. Both transport mechanisms were simulated in wxAMPS for *n*-Si(111)|CH_3_|*a*-TiO_2_ photoelectrodes. The tunneling simulation results in an immediate, exponentially decreasing current versus TiO_2_ thickness which did not match the experimental observation. The defect mediated transport mechanism shown in Figure 10(b), where holes move through a defect band in the band gap of the TiO_2_, was shown by wxAMPS to match the experimentally observed photocurrent versus thickness response.

#### 4.2.2. Non-leaky amorphous titanium dioxide

CaFe_2_O_4_ (CFO) is a promising p-type semiconductor to serve as a photocathode for H_2_-evolution in a PEC water-splitting system [91,92]. TiO_2_ has been introduced as a passivation layer on CFO and shown to improve the chemical stability of the material [84]. Despite improved stability, the onset potential of the photocathode upon TiO_2_ deposition is reduced. wxAMPS was used to determine the charge transport limitation behind the reduced onset potential and predict ways to improve the TiO_2_-coated CFO photocathode [84].

Modeling both the coated and uncoated CFO photocathode behaviors showed that the reduction in onset potential upon coating CFO with TiO_2_ was a consequence of photo-generated charge trapping in the TiO_2_. When the CFO/TiO_2_ photocathode is under irradiation from a full spectrum solar simulator, the wavelengths of light that exceed the band-gap of TiO_2_ result in photoexcitation of carriers within the TiO_2_ layer. These accumulate in surface trap states, resulting in an electric field in the TiO_2_ that reduces the V_oc_. The simulations predicted that the reduction in the onset potential could be avoided by utilizing a UV light filter and only exciting the CFO/TiO_2_ photocathode with visible light. Excluding the UV light, the TiO_2_ acts as a protective electron-selective contact between the CFO and the electrolyte and the trap states in the TiO_2_ do not influence the onset potential. The simulation results predicted an onset potential of ~1.9 V versus a reversible hydrogen electrode (RHE). This indicated that the V_oc_ of TiO_2_-coated CFO under longer wavelength irradiation should provide enough electrochemical potential to evolve H_2_ in a PEC water-splitting cell without an external applied voltage.

**Figure 11.**
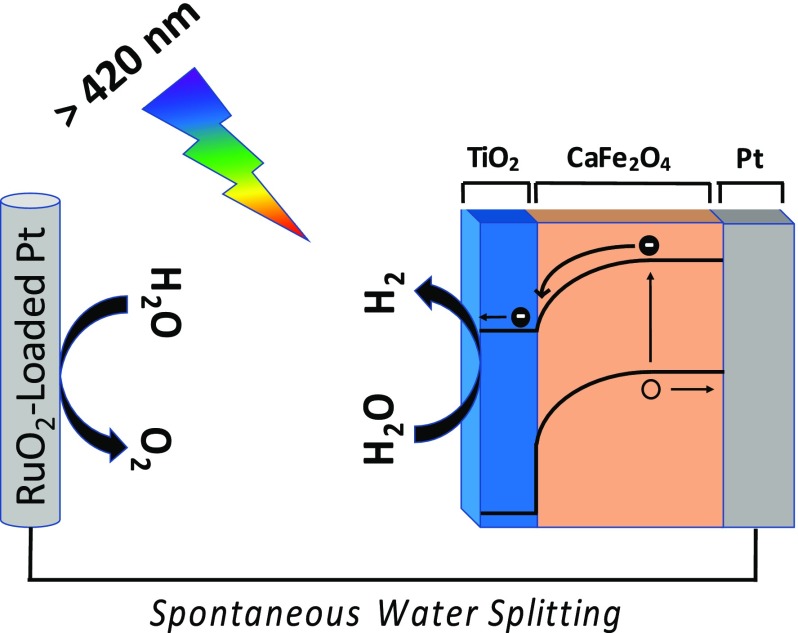
Schematic of a photoelectrochemical cell consisting of TiO_2_-coated/CaFe_2_O_4_ in series with RuO_2_-Loaded Pt. This cell split water spontaneously without an applied bias under visible light illumination. The cell was designed and optimized using wxAMPS simulations.

The simulation predictions were validated experimentally. First, the onset potential of the CFO/TiO_2_ photocathode under visible light irradiation was measured and found to be at minimum 1.6 V versus RHE. The actual onset potential could not be observed due to the onset of water electrolysis allowing current flow. The optimized TiO_2_-coated CFO under visible light irradiation had the most positive onset potential among the oxide photocathodes ever reported for PEC water-splitting, which was achieved by understanding the charge transport using wxAMPS. The TiO_2_-coated CFO photocathode was connected in series with a RuO_2_-loaded Pt system and under 470 nm excitation (visible light), evolution of H_2_ without an external applied bias was observed. A schematic of the device including the charge transport mechanism within the CFO/TiO_2_ photocathode is shown in Figure 11.

## 5. Future directions

Throughout this review, we have shown that density functional theory and finite-element device modeling are capable of providing insight into the charge transport characteristics of complex heterojunctions. Both models have been used to investigate charge transfer across heterojunctions consisting of metals, semiconductors, dielectrics, and electrolytes, including surfaces chemically functionalized with organic moieties. Predictions made using these computational tools have been demonstrated to be correct through subsequent experiments. The methods described can thus be used to significantly improve the performance of photoelectrochemical devices.

To further improve the predictive potential of the models, the two levels of theory must be connected as a functional multiscale device model. Ongoing work to demonstrate this connection is underway, using DFT to estimate model parameters to be used as inputs to wxAMPS. Then, the drift-diffusion model is used to predict the performance of the device based on the DFT estimations. This approach provides a predictive capability that is used to direct experimental approaches for controlling charge transfer across semiconductor surfaces. Further improvements to wxAMPS are also in progress incorporating more complex electrolyte models such as charge transfer from surface states.

## Disclosure statement

No potential conflict of interest was reported by the authors.

## Funding

This work was supported by the International Institute for Carbon Neutral Energy Research (WPI-I2CNER), sponsored by the Japanese Ministry of Education, Culture, Sports, Science and Technology and the National Science Foundation [grant number 1545907].
